# Can Chunk Size Differences Explain Developmental Changes in Lexical Learning?

**DOI:** 10.3389/fpsyg.2015.01925

**Published:** 2016-01-07

**Authors:** Eleonore H. M. Smalle, Louisa Bogaerts, Morgane Simonis, Wouter Duyck, Michael P. A. Page, Martin G. Edwards, Arnaud Szmalec

**Affiliations:** ^1^Psychological Sciences Research Institute, Université catholique de LouvainLouvain-la-Neuve, Belgium; ^2^Department of Experimental Psychology, Ghent UniversityGhent, Belgium; ^3^Department of Psychology, University of HertfordshireHatfield, UK

**Keywords:** Hebb repetition learning, language acquisition, lexical development, working memory, chunking

## Abstract

In three experiments, we investigated Hebb repetition learning (HRL) differences between children and adults, as a function of the type of item (lexical vs. sub-lexical) and the level of item-overlap between sequences. In a first experiment, it was shown that when non-repeating and repeating (Hebb) sequences of words were all permutations of the same words, HRL was slower than when the sequences shared no words. This item-overlap effect was observed in both children and adults. In a second experiment, we used syllable sequences and we observed reduced HRL due to item-overlap only in children. The findings are explained within a chunking account of the HRL effect on the basis of which we hypothesize that children, compared with adults, chunk syllable sequences in smaller units. By hypothesis, small chunks are more prone to interference from anagram representations included in the filler sequences, potentially explaining the item-overlap effect in children. This hypothesis was tested in a third experiment with adults where we experimentally manipulated the chunk size by embedding pauses in the syllable sequences. Interestingly, we showed that imposing a small chunk size caused adults to show the same behavioral effects as those observed in children. Departing from the analogy between verbal HRL and lexical development, the results are discussed in light of the less-is-more hypothesis of age-related differences in language acquisition.

## Introduction

In this paper, we will investigate whether the Hebb learning effect in immediate serial recall (Hebb, 1961) can shed light on whether children learn verbal sequences differently from adults. It is assumed that children learn complex structures by chunking them into small units, and that this could provide them with a cognitive advantage when learning novel word-forms (c.f., the less-is-more hypothesis; Newport, 1990; Elman, 1993). Hebb repetition learning (HRL) is a well-known sequential-learning paradigm that is assumed to rely on the same cognitive resources as word-form learning (Page and Norris, 2009a,b). In line with the less-is-more hypothesis, therefore, we hypothesize that children chunk Hebb sequences in smaller units than do adults, resulting in stronger Hebb-learning effects. Previous Hebb learning studies found weak Hebb effects in children (Mosse and Jarrold, 2008; Archibald and Joanisse, 2013; Hsu and Bishop, 2014; Bogaerts et al., in press). It should be noted, however, that previous studies (a) employed exclusively sequences of lexical items (i.e., word or digit sequences) and (b) tested HRL under circumstances in which all sequences, whether repeated or not, were permutations of the same small set of items (i.e., conditions of “item-overlap”). This does not resemble naturalistic word-form learning. In two experiments, we will address both of these issues by directly comparing children and adults on a Hebb-learning task with overlapping and non-overlapping sequences, first using lexical items (i.e., sequences of words, Experiment 1) and then using sub-lexical items (i.e., sequences of syllables, Experiment 2). In a third experiment, we will investigate whether we can induce “child-like” behavior in adults by encouraging them to chunk syllable sequences in small units. Before describing these experiments, we will sketch out the theoretical background in more detail.

### Starting small in language development

It is widely accepted that sensitivity to language input varies as a function of age, including the consensus that language acquisition should preferably take place before adolescence to achieve native-like performance (Penfield and Roberts, 1959; Lenneberg, 1967; Johnson and Newport, 1989; Pinker, 1994; Birdsong, 2006; Singleton, 2007). However, the exact nature, cause and magnitude of this *sensitive period* phenomenon in language learning remains an issue of wide controversy (Hyltenstam and Abrahamsson, 2003; Birdsong, 2006; DeKeyser, 2013), to the extent that the journal *Science*, in its 125th anniversary edition, labeled the sensitive-period hypothesis as one of the most fundamental yet unresolved questions in human science (Kennedy and Norman, 2005). According to one language acquisition theory, maturational constraints on language learning are explained by constraints on cognitive resources in childhood. Newport's (1990) *less-is-more* theory of language development posits that children are more successful at language acquisition than adults because their limited working memory capacity forces them to process a truncated portion of the input, allowing them better to analyze their language into its smallest component structures rather than memorizing larger, misleading chunks of input (Newport, 1990; Elman, 1993; Erickson and Thiessen, 2015). Elman (1993) tested this idea by training a simple recurrent network (SRN) to learn complex language structures. Under normal conditions, the network was unable to learn the sequential regularities of an artificial language. But when Elman simulated children's working memory limitations and the network was exposed to a staged input (item-by-item) instead of the entire structure at once, the neural network's performance improved. The empirical evidence gathered from human participants is, however, still far from conclusive (Conway et al., 2003; Lai and Poletiek, 2011).

### Sequential learning in novel-word acquisition

Sequential learning, defined as the ability to encode and represent the order of discrete elements occurring in a sequence, is an important aspect of human cognition and skill learning (Conway and Christiansen, 2001). Sequential inputs are typically *chunked* into units or subsequences of items (Lashley, 1951), recombined and, hence, memorized to acquire a full representation of the sequential structure (Brooks and Vokey, 1991; Saffran, 1996, 2001; Perruchet and Pacton, 2006; Lafond et al., 2010). It is generally accepted that several aspects of language learning and processing are sequential in nature (Saffran, 1996, 2001; Conway and Christiansen, 2001; Lafond et al., 2010; Hsu and Bishop, 2014). For example, sequences of phonemes form words and words in turn are sequentially aligned to form legal grammatical phrases (Pinker, 1994). An important source of evidence for sequential learning in language acquisition is Saffran's statistical learning approach in young infants (Saffran, 1996, 2001). In her studies, infants were exposed to a continuous speech stream, which consisted of three three-syllable “pseudowords” that were repeated in random order (e.g., *pabiku, golatu*, and *daripo* in *pabikugolatudaropigolatupabikudaropi)*. In a subsequent test, infants turned their heads more often and looked longer to the “pseudowords” (e.g., golatu) compared with part-words (i.e., sequences spanning a word-boundary, e.g., bikugo). This demonstrates that infants can segment a continuous speech input stream on the basis of the probability of co-occurrence between the syllables (i.e., the transitional probabilities). It has been argued that this sensitivity to transitional probabilities is a reflection of underlying chunking mechanisms according to which adjacent syllables are grouped into chunk representations that receive activation every time they are encountered. Within this view, representations of groupings across word boundaries will show less (re)activation because they are re-encountered less frequently during exposure; hence they will suffer in competition with representations of groupings within word boundaries (see PARSER, Perruchet and Vinter, 1998; and, Extraction and Integration Framework, Erickson and Thiessen, 2015). This means that whereas learners may appear to be sensitive to transitional probabilities, they are actually storing chunks of the input stream, which—owing to interference in memory—are biased toward those statistically coherent chunks that are frequently encountered during exposure (Erickson and Thiessen, 2015). This chunking hypothesis offers a different perspective on the way we learn sequences, compared with an explanation based solely on transition probabilities (Jones, 2012).

Further experimental evidence for the role of sequential learning in language acquisition comes from research within the Hebb repetition-learning paradigm (Hebb, 1961; Mosse and Jarrold, 2008; Lafond et al., 2010). When, unannounced to participants, one particular sequence of items (i.e., letters, phonemes) is repeated in the same order during an immediate serial recall task, performance for the repeating sequence (often called a Hebb sequence) improves relative to non-repeating (filler) sequences (Hebb, 1961). This finding is known as the Hebb repetition effect (HRE), and reflects the gradual transfer of newly acquired serial-order information from short-term to long-term memory. Learning in the Hebb repetition task can be considered to be implicit, as it occurs even without explicit awareness of the repetition (Gagnon et al., 2004, 2005; Couture and Tremblay, 2006; Guérard et al., 2011). It has been hypothesized that the HRE relies on the same underlying mechanisms as word-form learning. In the model of Page and Norris (2009a,b), a new word-form is conceived as a familiarized sequence of sub-lexical components (e.g., *lo*-*fo*-*du)*. Repetitive learning of a syllable sequence in a Hebb repetition experiment is, according to this hypothesis, functionally equivalent to acquiring a corresponding novel word-form (e.g., “lofodu”). Previous work on the Hebb paradigm corroborated this hypothesis by the use of subsequent lexicalization tasks (Szmalec et al., 2009, 2012). Participants recalled sequences of nine CV syllables, grouped by pauses into three sets of three syllables, for immediate serial recall. One repeating (Hebb) sequence contained nonsense syllable groups that were neighbors of existing base-words (e.g., la-va-bu, sa-fa-ro, no-ma-du, that are close to the existing Dutch words lavabo, safari, and nomade). After learning and following an offline consolidation period of several hours, lexical decision and pause detection tasks showed higher reaction times for existing words that, by hypothesis, had acquired new competitors in the lexicon as a result of Hebb learning, slowing down their lexical decision and pause detection. This indicates that novel entries corresponding to repeated syllable sequences are created in the mental lexicon through the process of repetitive serial-order (Hebb) learning. An increasing amount of experimental work is consistent with this hypothesis (Mosse and Jarrold, 2008; Gaskell and Ellis, 2009; Majerus and Boukebza, 2013; Page et al., 2013; Hurlstone et al., 2014), and has extended these findings toward developmental samples (Mosse and Jarrold, 2008) and samples with developmental language disorders (Szmalec et al., 2011; Archibald and Joanisse, 2013; Hsu and Bishop, 2014; Bogaerts et al., 2015, in press).

### Item-overlap effects in the Hebb repetition paradigm

As briefly mentioned above, Page and Norris (2009a,b) described a unifying model that accounts for the HRE and the generic long-term learning of sequences, such as phonological word-forms. According to their model, the learning of a particular sequence (e.g. *lo-fo-du-be-ka-li-da-mu-vo*) comprises the allocation of one or more new chunk representations that are activated by subsequent presentations of the learned sequence, hence enhancing recall performance as Hebb learning proceeds. The occurrence of a Hebb effect is a result of two important assumptions. First, that any novel sequence that occurs during the Hebb task will activate a number of previously uncommitted chunk representations. One of these will become *engaged* in response to that sequence, and a *commitment* starts in learning that sequence. Second, as a result of this first-trial learning, the engaged chunk representation will be more strongly activated on several subsequent presentations (i.e., repetitions) of the same sequence. As learning proceeds, the chunk representation becomes more order-sensitive and a competitive process starts during which chunk representations start *competing* with each other to represent a given stimulus sequence.

The Page and Norris (2009a,b) model offers an explanation for several findings with the Hebb repetition paradigm, and explicitly addresses the hypothesis that HRE is underpinned by the same mechanisms as word-form learning. For example, the model can explain (a) why HRL still occurs when repetitions are spaced further apart (e.g., every sixth trial, or even every twelfth trial, instead of every third trial), (b) why learning of multiple Hebb sequences is possible when they are presented in interleaved fashion (e.g. one Hebb sequence is presented on trials 2, 6, 10 etc. and another Hebb sequence is presented on trials 4, 8, 12 etc. with filler sequences as non-repeating, intervening trials), and (c) how sequences can still be represented in memory 3–4 months after initial learning. All this is encouraging evidence for the hypothesis that the Hebb effect is a laboratory analog of the word-form learning process, given that novel word-form representations are unlikely to be closely spaced in daily life or to occur in the absence of other competing word-forms. Interestingly, Mosse and Jarrold (2008) further also found that the magnitude of Hebb learning using both verbal (i.e., sequences of digit words) and visuospatial stimuli, correlated significantly with non-word (sublexical) learning in a paired-associate learning task, when testing young children. This provides further evidence for the hypothesis that Hebb learning, and more precisely the core ability to represent and learn serial-order information across modalities, taps into similar mechanisms as does word-form learning (Szmalec et al., 2009, 2012).

Page et al. (2013) further developed their model of the Hebb effect by manipulating the overlap between item-sets used in repeating and non-repeating sequences in the Hebb task. Overall, they observed reduced Hebb learning in adults when all sequences were permutations of the same items. Remember that, according to their model, Hebb learning requires that every distinct sequence in the task (including every filler sequence) engages a previously uncommitted chunk-node on its first presentation. In other words, every sequence will be partly learned on its first presentation—this is a logical requirement, given that it is not known in advance which sequences will subsequently repeat and which will not. When all sequences (repeating and fillers) are derived from the same item-set, therefore, by the time that the first repetition of a Hebb sequence occurs, there will be several engaged chunk representations, of which one is engaged to the Hebb sequence and all the others are engaged to perfect anagrams of this Hebb sequence (since the filler sequences are permutations of the same items—this explanation assumes, for simplicity, that each sequence is learned as a single chunk, an assumption that is relaxed below). As a result, early in learning, when representations are not yet very order-selective, the chunk representations of all filler sequences will substantially co-activate in response to presentation of the repeating (Hebb) sequence. This mass co-activation of chunk units representing filler sequences makes it harder to identify the chunk unit that is committed to the repeating Hebb sequence. As a result, by hypothesis, learning of that repeating sequence is slower.

### Hebb learning in children

Although Hebb representations are learned relatively fast and in a manner that is stable across time, HREs observed in children appear to be relatively weak (Mosse and Jarrold, 2008; Hsu and Bishop, 2014; Bogaerts et al., in press). This is surprising because, when considering the ease with which children acquire novel word-forms from linguistic input in their environment, one might anticipate that children would be good or even better at Hebb learning than adults. However, the few studies that have investigated Hebb learning in children have used sequences of digits or words instead of sequences of the phonemes or syllables that constitute the true sublexical basis of novel word-forms. Furthermore, in previous Hebb learning studies, Hebb and filler sequences showed full item-overlap, which is not the case for real-world word-form acquisition (see also Page et al., 2013); this might have contributed to children's weak Hebb effects, consistent with the way in which it contributed to a weakening in adults' HRL (see Page et al., 2013).

### The current study

Using the Hebb repetition paradigm, the present work aims to clarify the cognitive origins of novel word-form learning in adults and children, within a model that explicitly links word-form learning to the establishment of chunk representations in memory (Miller, 1956; Servan-Schreiber and Anderson, 1990; Jones, 2012). In the first two experiments, we address the issues of (sub)lexical stimulus material and item-overlap, which may account for children's weak Hebb learning effects in previous studies. Experiment 1 was designed to estimate the effect of item-overlap between the filler and Hebb sequences in adults and children, using sequences of lexical stimuli. We expected to see an item-overlap effect in adults (similar to Page et al., 2013) and also in children. In Experiment 2, the same manipulations were adopted in a Hebb-learning experiment using sequences of sublexical materials (i.e., syllables). We assume that Hebb-sequence learning of sublexical items is more comparable to naturalistic word-form learning and therefore sublexical materials offer us a more valid means of comparing verbal sequence (or word-form) learning in adults and children. We anticipated that children would show stronger Hebb learning effects compared with adults, but only when there is no item-overlap. In order to estimate more directly whether the age-related differences in Hebb learning in Experiment 2 reflect chunking differences, we conducted Experiment 3. In this final experiment, we inserted pauses in the verbal sequences in order to investigate whether we can induce “child-like” behavior in adults by encouraging them to chunk the Hebb sequences into smaller units, as an approximate simulation of children's chunking preferences (Newport, 1990; Elman, 1993; Jones, 2012).

## Experiment 1

**Figure 1:**
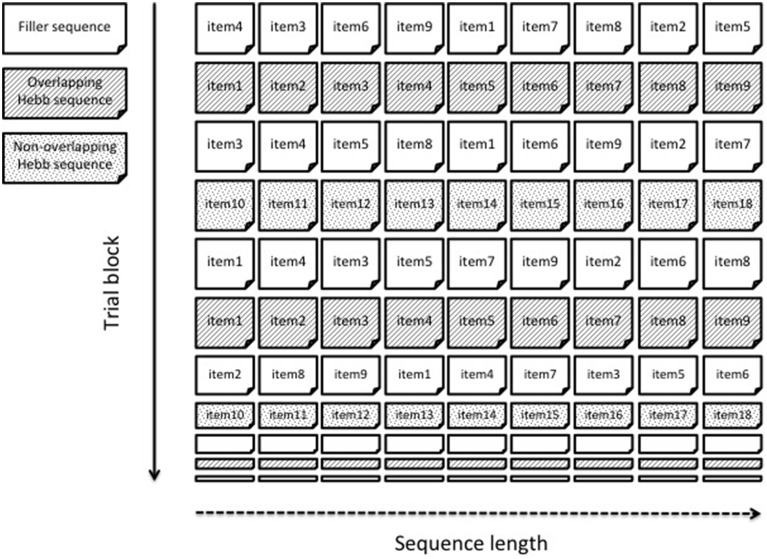
**An example of a within-block overlap manipulation**. Two different Hebb sequences are presented within one learning block. The overlapping Hebb sequence contains the same items as the intervening filler sequences. The non-overlapping Hebb sequence contains different items. Only the first 10 trials are shown.

In this experiment, we aimed to replicate Page et al.'s (2013) findings of reduced HRL in adults as a result of item-overlap between sequences, and to extend these findings to children. The same type of material was used as in Page et al. (2013), that is, sequences of one-syllable words. Unlike Page et al. (2013), and to make the Hebb task child-friendly, we presented word sequences auditorily and participants were required to recall the sequence orally. Moreover, overlap of items was manipulated within one Hebb learning block, instead of between separate blocks as in Page et al. (2013). To this end, two Hebb sequences were presented in an interleaved fashion, with one Hebb sequence being a permutation of the same items as the non-repeating filler sequences and the second Hebb sequence being constructed from different items. This within-block design is illustrated in Figure 1. Including both Hebb sequences in the same block allows a direct comparison between overlapping and non-overlapping Hebb sequences, ensuring that the overlap effect is not confounded with baseline differences in filler performance (which in a mixed design is the same for both conditions of overlap). Children and adults were directly compared. Overall we predicted that learning (i.e., improvement in recall performance across trials) for the overlapping Hebb sequence would be weaker compared with learning for the non-overlapping Hebb sequence, independently of the age group.

### Participants

In total, 40 twelve-year old children and 39 adults took part in the study. All children were recruited from four different schools in and around Brussels, the capital city of Belgium. Adults were recruited by means of advertising. We excluded participants who were diagnosed with dyslexia or dyscalculia (*n* = 4 in the children group) based on earlier evidence that HRL is impaired in dyslexia (Szmalec et al., 2011; Bogaerts et al., 2015). As a result, 36 children (mean age 11.7 ± 0.6 SD; 8F/28M) and 39 adults (mean age 31.4 years ± 12.4 SD; 21F/18M) were included for analysis. All participants were French-speaking^1^. None of them suffered from any developmental, psychiatric or neurological disorder. All participants gave informed consent (parental consent was obtained for children). Neither children nor adults received any financial compensation for their participation. The Université catholique de Louvain, Faculty of Psychology Ethics Commission approved the experiment.

### Materials

Sequences of single-syllable French nouns, all with an age of acquisition (AoA) lower than 6 years, were presented to the participants for immediate serial recall. The stimuli can be found in Table 1. We adjusted the length of the sequences to the mean span of the age group and increased this by two more items to avoid ceiling effects in HRL (resulting sequence-lengths were eight items for children and nine items for adults). A pilot study on two 12-years-olds and two adults was performed to confirm that the two groups were tested at a comparable performance level (i.e., similar filler performance across trials). To create the sequences, two item sets (A and B) of nine words were generated by using Lexique 3.80 (New et al., 2004), and matched for AoA (*F* < 1 for both groups) (see Table 1). For the 12-years-olds, the word *doigt* from set A and *feu* from set B were excluded to obtain 8-item sequences that were matched on mean AoA to the adult's 9-item sequences. Ten different sequence orders were created from each item set and counterbalanced across our two Hebb conditions [i.e., overlapping Hebb condition (H*o*) vs. non-overlapping Hebb condition (H*n*)] to avoid stimulus-specific effects. The filler sequences contained the same sequence-items as the overlapping Hebb sequence, but in a different order. The order of words within the filler sequences was determined randomly by using the E-prime 2.0 software (Psychology Software Tools, Pittsburgh, PA) algorithm (Schneider et al., 2002). The Hebb learning block consisted of 32 sequences in total, which were all presented for immediate recall. Both the H*o* and H*n* sequences were mixed within the same block and were repeated on every fourth trial, that is, eight times in total, interspersed with a total of 16 filler sequences.

**Table 1:** **Stimuli material for Experiment 1**.

**Set A**		**Set B**		
**CV**	**AoA**	**CV**	**AoA**	
CHAT	3.80	PIED	3.60	*Children + Adults*
OEIL	4.10	RUE	5.20	*Children + Adults*
OEUF	4.50	BOUE	5.80	*Children + Adults*
BEAU	4.50	PLUIE	4.40	*Children + Adults*
DOUX	5.10	OIE	5.60	*Children + Adults*
TRAIN	5.10	JOUR	4.70	*Children + Adults*
MAIN	3.60	NUIT	4.10	*Children + Adults*
BRAS	4.20	LOUP	4.70	*Children + Adults*
DOIGT	3.70	FEU	4.90	*Adults*

*Eight and nine single-syllable words were used for 12-years-olds and adults respectively. Age of Acquisition (years) for each word is reported*.

### Procedure

The experiment started with a familiarization phase in which participants listened to each word that would be used in the task. They were instructed to repeat the words out loud and were corrected if necessary. We ensured that all words were known by the participants by asking them to define each word separately. All words were recorded by a female voice and presented auditory at 60 dB using Sennheizer HD265-1 headphones. The experiment was presented electronically using the E-Prime 2.0 software (Psychology Software Tools, Pittsburgh, PA) running on a Windows PC. The words were presented one at a time for 750 ms with an inter-stimulus interval of 250 ms. The Hebb learning procedure was similar to that in previous studies (Page et al., 2006, 2013; Szmalec et al., 2009, 2012). The task always started with presentation of three filler sequences followed by one of the Hebb sequences, one filler sequence and the other Hebb sequence (i.e., f, f, f, H*n*, f, H*o*, f, H*n*, f, H*o*, … or f, f, f, H*o*, f, H*n*, f, H*o*, f, H*n*,…), counterbalanced across participants. The two first filler sequences were introduced as a practice. Immediately after sequence presentation, a recall screen was presented with a question mark signaling that the participants had to recall the CVs in the same order as presented. They were allowed to say “blank” when they forgot a word at a particular serial position. The Hebb learning task lasted approximately 30 min.

### Results

**Figure 2:**
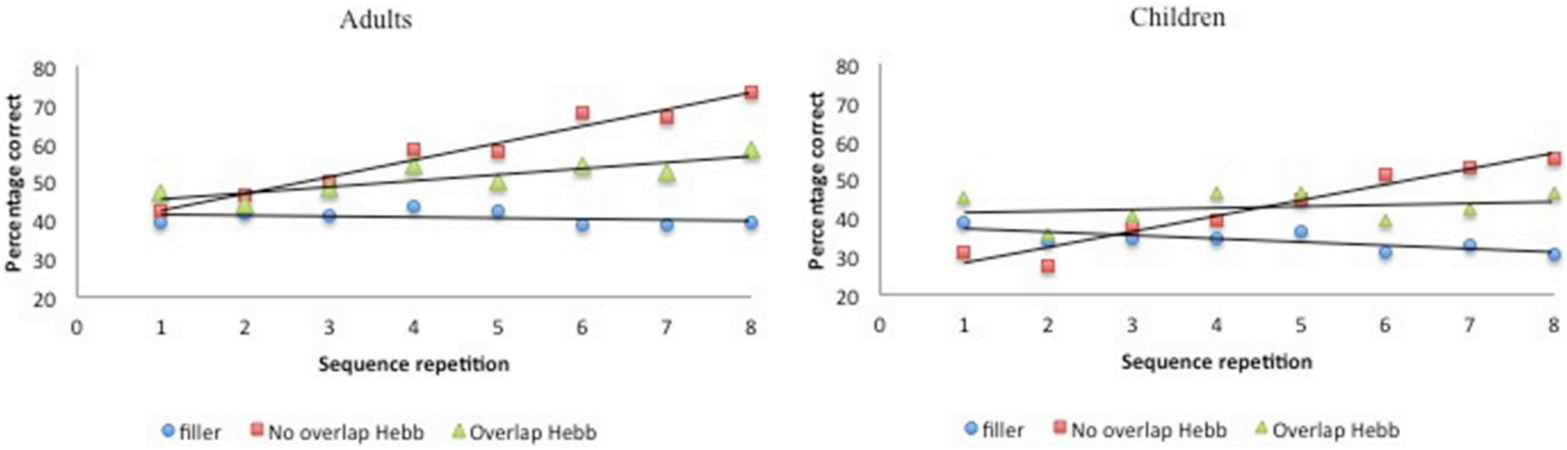
**Performance (percentage of correct scores) as a function of Sequence type (filler vs. Hebb non-overlap vs. Hebb overlap) and Sequence repetition (1–8) in both children and adults, Experiment 1**. **Left panel**: performance for adults. **Right panel**: performance for children.

We scored Hebb recall performance using McKelvie's (1987) scoring method. This method takes into account both the position and the serial order of recalled items. In a first step, the number of items is counted that are in the correct position from left to right up to the first error. Secondly, the same step is repeated from right to left up to the first error. After this, the number of items in any correct sequence of two or more items between the first error from the left and the first error from the right is counted. Finally, any other items that occur in the correct position from left to right are counted^2^. The maximal possible recall score using this procedure was 8 for the children (i.e., for sequences of 8 items) and 9 for the adults (i.e., for sequences of 9 items). Recall performance for the filler sequences was averaged across two consecutive filler trials to obtain an equal number (i.e., eight) of filler and Hebb repetition scores. An arcsine square root transformation was completed on all percent scores in order to transform the fixed-limit distribution of percentages to a normal distribution appropriate for statistical analyses (Archibald and Joanisse, 2013). For clarity, all descriptive statistics presented in the tables and figures represent the untransformed percentage of correct scores. The data are plotted in Figure 2.

**Figure 3:**
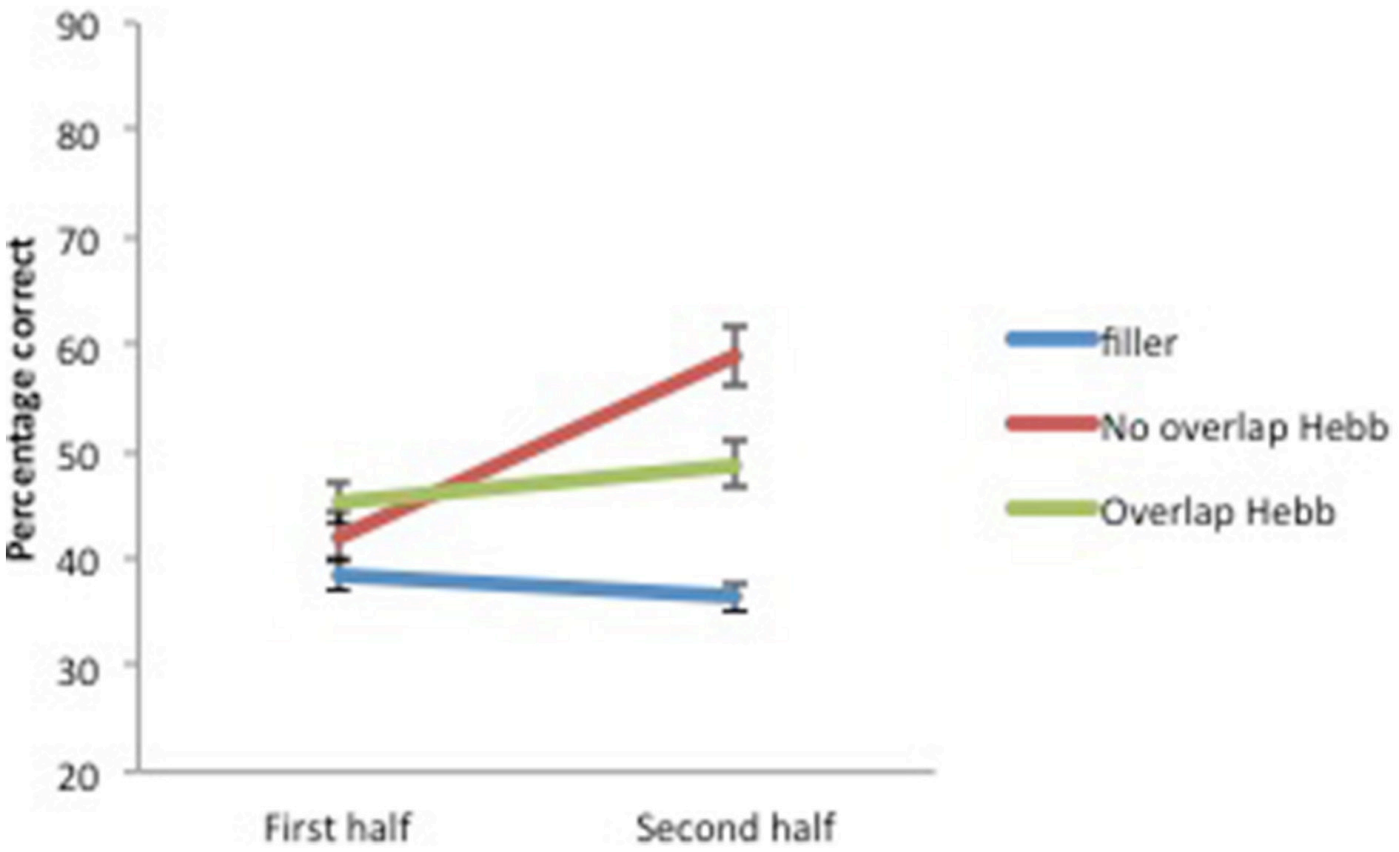
**Mean percentage of items correctly recalled (with standard errors) for Hebb and filler sequences by sequence Halves, Experiment 1**.

Recall accuracy was analyzed using a 2 (Group: children vs. adults) × 2 (Half: first vs. second) × 3 (Sequence type: filler vs. Hebb non-overlap vs. Hebb overlap) repeated measures ANOVA. In order to evaluate implicit learning in the Hebb task, we employed the procedure adopted by Mosse and Jarrold (2008) as well as Archibald and Joanisse (2013) that involves comparing performance on the first and second halves of each sequence type (for similar procedure, see also Turcotte et al., 2005)^3^. While a main effect of Sequence type in favor of the Hebb sequence might provide some evidence of learning that sequence, only the demonstration of improvements in performance for repeated Hebb sequences, relative to the baseline filler sequences, can be taken as an indication of implicit learning. Thus, an interaction between Sequence type and Half due to higher scores on the Hebb sequences for the second half of the trials would provide evidence of Hebb learning (Archibald and Joanisse, 2013). The ANOVA revealed a significant main effect of Group [*F*_(1, 73)_ = 12.89, *p* < 0.001, $n_{p}^{2}=0.15$] with adults showing higher recall scores than children (49.66 ± 1.17_*SE*_ vs. 39.58 ± 1.32_*SE*_). There was also a significant main effect of Half [*F*_(1, 73)_ = 37.24, *p* < 0.001, $n_{p}^{2}=0.34$], such that recall scores for the second half of the repetitions were higher than recall scores for the first half of the repetitions (47.75 ± 1.40_*SE*_ vs. 41.88 ± 1.13_*SE*_), and a significant main effect of Sequence type [*F*_(2, 146)_ = 19.61, *p* < 0.001, $n_{p}^{2}=0.21$]. Comparisons revealed higher recall scores for the non-overlapping Hebb sequence (50.42 ± 1.93_*SE*_) compared with the overlapping Hebb sequence (46.59 ± 1.45_*SE*_) [*F*_(1, 146)_ = 19.35, *p* < 0.00, $n_{p}^{2}=0.12$] and the filler sequence (37.45 ± 1.02_*SE*_)[*F*_(1, 146)_ = 147.09, *p* < 0.001, $n_{p}^{2}=0.50$]. Recall scores for the overlapping sequence were also significantly higher than for the filler sequence [*F*_(1, 146)_ = 59.75, *p* < 0.001, $n_{p}^{2}=0.29$]. Further, there was a significant interaction between Half and Sequence type [*F*_(2, 146)_ = 47.74, *p* < 0.001, $n_{p}^{2}=0.40$]. This did not differ significantly between groups [*F* < 1]. The significant two-way interaction is illustrated in Figure 3. Planned comparisons of the significant interaction between Half and Sequence type revealed a significant increase across halves for the non-overlapping Hebb sequence [*F*_(1, 146)_ = 141.28, *p* < 0.001, $n_{p}^{2}=0.49$]. Comparable contrasts for the other sequences were non-significant. During the first half of the task, recall was higher for both the overlapping and non-overlapping Hebb sequence compared with the filler sequences [*F*_(1, 146)_ = 49.56, *p* < 0.001, $n_{p}^{2}=0.25$ and *F*_(1, 146)_ = 3.86, *p* < 0.051, $n_{p}^{2}=0.03$, respectively]. There was no difference between the two Hebb sequences. During the second half of the task, as during the first half, recall was higher for both the overlapping and non-overlapping Hebb sequence compared with the filler sequences [*F*_(1, 146)_ = 61.55, *p* < 0.001, $n_{p}^{2}=0.30$ and *F*_(1, 146)_ = 230.65, *p* < 0.001, $n_{p}^{2}=0.61$ respectively]. However, the non-overlapping Hebb sequence scored significantly higher than the overlapping Hebb sequence [*F*_(1, 146)_ = 66.38, *p* < 0.001, $n_{p}^{2}=0.31$].

### Discussion

In Experiment 1, we manipulated overlap between the lexical items of Hebb and filler sequences. The results showed that although recall was significantly better for both the non-overlapping and overlapping Hebb sequences compared with the filler sequences, the non-overlapping Hebb sequence showed the strongest learning pattern. This was indicated by the significant improvement across halves and the better recall during the second half of the task. These results are similar to what was found with adults in Page et al. (2013), and these observations can be extended, for the first time, to younger learners. Note that in the current study a different presentation modality (auditory) and recall modality (oral) were used, and that overlap was manipulated within the same Hebb learning block, compared with Page et al. (2013). This shows that the overlap effect is robust. Importantly for the current study, no interactions with group were found. This indicates that HRL and its sensitivity to overlap can be generalized across development. Note that recall during the first half of the task was higher for Hebb sequences compared with filler sequences. We argue that this can be explained by the rapid memorization of the Hebb sequence during the first four repetitions (see Figure 2).

In the next experiment, we aimed to test the effect of item-overlap in children and adults when using sublexical items (i.e., sequences of syllables). Because children are assumed to show very strong word-learning skills throughout childhood (e.g., Pinker and Jackendoff, 2005), we predicted that the children in the current experiment would acquire the sublexical sequences, which are functionally equivalent to novel words, more rapidly than the adults and that this would be reflected in a stronger Hebb-learning effect. In addition, in line with Page et al.'s (2013) theoretical framework, and in an attempt to offer an explanation for weak HRE with children in previous studies, we predicted that children would, if anything, be more significantly affected by item-overlap during learning.

## Experiment 2

### Participants

The same participants took part as in Experiment 1.

### Materials and procedure

Exactly the same procedure was used as in Experiment 1, except for the items, which were nonsense syllables instead of words. All syllables had a consonant-vowel structure (CV). Again the length of the sequences was adjusted to the memory span of the age group, increased by two syllables and piloted in two children and adults. Two sets (A and B) of nine syllables were generated by the use of WordGen (Duyck et al., 2004) and matched for biphone frequency (*F* < 1 for both groups). Both item sets are presented in Table 2. For the 12-year-old children, the CVs *xu* from set A and *wu* from set B were excluded to match sequences used in children with sequences used in adults (on mean biphone frequency). We ensured that consecutive phonemes could not sound like existing words in French (e.g., *cave* or *colis)*. All CVs were recorded by the same female voice as in Experiment 1 and presented auditorily at 60 dB using Sennheizer HD265-1 headphones. The CVs were presented for 500 ms with an inter-stimulus interval of 500 msec. The Hebb learning task lasted approximately 30 min.

**Table 2:** **Stimuli material for Experiment 2**.

**Set A**		**Set B**		
**CV**	**Biphone**	**CV**	**Biphone**	
TI [ti]	3440	LI [li]	2843	*Children + Adults*
RI [ri]	3880	NA [na]	1262	*Children + Adults*
JA [a]	981	GU [gy]	173	*Children + Adults*
MU [my]	438	CO [ko]	1388	*Children + Adults*
SO [so]	155	FI [fi]	1142	*Children + Adults*
VE [v 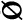 ]	765	PE [p 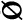 ]	960	*Children + Adults*
BE [b 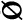 ]	354	ZE [z 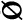 ]	631	*Children + Adults*
KA [ka]	2251	DA [da]	497	*Children + Adults*
XU [ksy]	197	WU [wy]	3	*Adults*

*Eight and nine syllables were used for 12-years-olds and adults respectively. French biphone frequency for each syllable is reported*.

### Results

**Figure 4:**
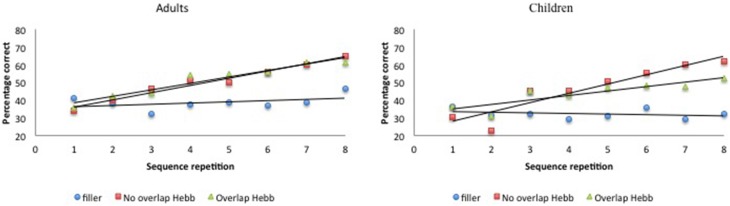
**Performance (percentage of correct scores) as a function of Sequence type (filler vs. Hebb non-overlap vs. Hebb overlap) and Sequence repetition (1–8) in both children and adults, Experiment 2. Left panel:** performance for adults. **Right panel**: performance for children.

**Figure 5:**
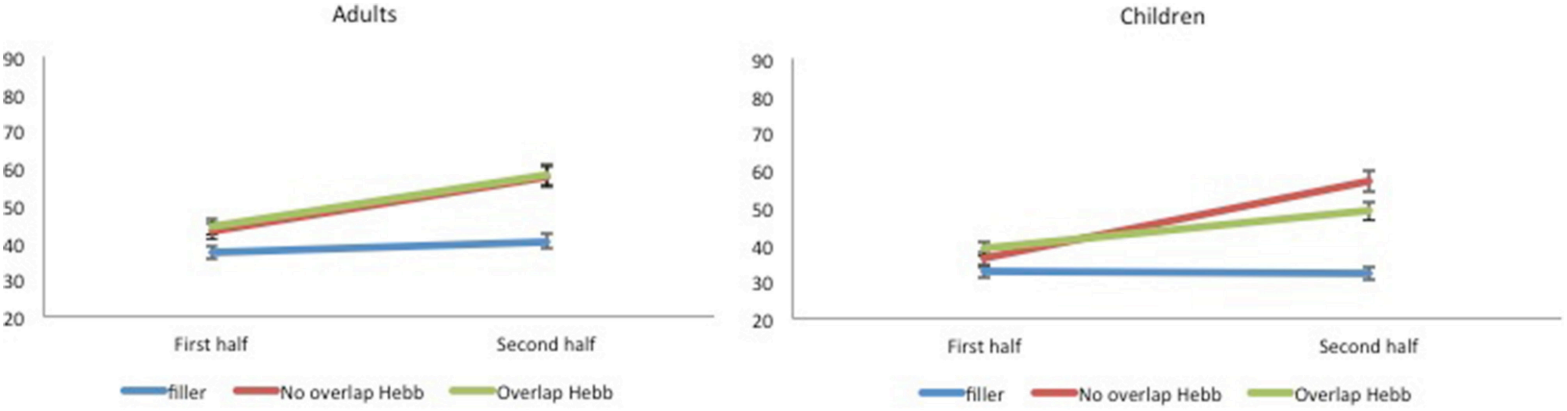
**Mean percentage of items correctly recalled (with standard errors) for Hebb and filler sequences by sequence Halves, in both children and adults, Experiment 2. Left panel:** performance for adults. **Right panel**: performance for children.

Again, McKelvie scoring was used to obtain immediate serial recall scores. The data are plotted in Figure 4. For each participant, the percentage correct scores were averaged across the first four and last four sequence repetitions, to obtain two halve scores, and transformed using arcsin square root transformation. The transformed scores were entered into a 2 (Group: children vs. adults) × 2 (Half: first vs. second) × 3 (Sequence type: filler vs. Hebb non-overlap vs. Hebb overlap) repeated measures ANOVA, as before. This yielded no significant effect of Group [*F*_(1, 73)_ = 2.32, *p* = 0.13, $n_{p}^{2}=0.03$]. There was a significant main effect of Half [*F*_(1, 73)_ = 93.66, *p* < 0.001, $n_{p}^{2}=0.56$] such that recall scores for the second half of the repetitions was higher than recall scores for the first half of the repetitions (48.22 ± 1.39_*SE*_ vs. 38.15 ± 1.05_*SE*_), and a significant main effect of Sequence type [*F*_(2, 146)_ = 26.46, *p* < 0.001, $n_{p}^{2}=0.27$]. Comparisons revealed better recall for the non-overlapping Hebb sequence (47.95 ± 1.73_*SE*_) compared with the filler sequences (34.80 ± 0.99_*SE*_)[*F*_(1, 146)_ = 122.74, *p* < 0.001, $n_{p}^{2}=0.46$], and better recall for the overlapping Hebb sequence (46.80 ± 1.64_*SE*_) compared with the filler sequences [*F*_(1, 146)_ = 88.71, *p* < 0.001, $n_{p}^{2}=0.38$]. There were no differences between the two Hebb sequences. Crucially, there was a significant interaction between Half and Sequence type [*F*_(2, 146)_ = 25.54, *p* < 0.001, $n_{p}^{2}=0.25$], that in turn interacted significantly with Group [*F*_(2, 146)_ = 4.34, *p* < 0.01, $n_{p}^{2}=0.13$]. This three-way interaction is illustrated in Figure 5. Planned comparisons within both groups revealed a significant non-overlapping Hebb effect (i.e., the different improvement across halves between filler and non-overlapping sequences) for children [*F*_(1, 146)_ = 41.31, *p* < 0.001, $n_{p}^{2}=0.22$], and adults [*F*_(1, 146)_ = 10.83, *p* < 0.01, $n_{p}^{2}=0.07$]. This non-overlapping Hebb effect was significantly larger for children compared to adults [*F*_(1, 146)_ = 5.55, *p* < 0.05, $n_{p}^{2}=0.04$]. Further comparisons revealed the presence of an overlapping Hebb effect in both children [*F*_(1, 146)_ = 8.00, *p* < 0.01, $n_{p}^{2}=0.05$] and adults [*F*_(1, 146)_ = 11.97, *p* < 0.001, $n_{p}^{2}=0.08$]. This did however not differ between groups, *F* < 1. Children showed a significantly lower improvement across halves for the overlapping Hebb sequence compared with the non-overlapping Hebb sequence [*F*_(1, 146)_ = 12.95, *p* < 0.01, $n_{p}^{2}=0.08$]. There was no such difference for adults, *F* < 1. Children and adults did not differ on differences across halves for the filler sequences, *F* < 1.

### Discussion

In Experiment 2, Hebb sequences were made of nonsense CV syllables instead of existing words. While children showed reduced Hebb learning due to item-overlap with filler sequences, surprisingly no such overlap effect was observed in adults. Although children (and adults) were still able to learn the overlapping sequence, as reflected by the improvement across halves (in contrast to Experiment 1), children showed less improvement on the overlapping compared with the non-overlapping Hebb sequence. Finally and very importantly, we observed a larger HRE in children compared with adults for non-overlapping syllable sequences.

In an attempt to explain what is driving the different results for item-overlap obtained for children vs. adults in the current experiment, we considered some recent simulation work on children's non-word (sublexical) repetition behavior using a computational instantiation of the chunking hypothesis, that is, the Elementary Perceiver and Memorizer (EPAM) (Feigenbaum and Simon, 1984; Jones, 2012). Overall, the chunking hypothesis suggests that repeated exposure to a stimulus set, for example, a sequence of phonemes or syllables, leads to the stimuli being represented in larger and larger chunks (Miller, 1956). Chunking may be very beneficial when one considers that short-term memory has a limited capacity. Only information that requires less than 2 s to process can be reliably stored in working memory (see Jones, 2012, using an approximation from Baddeley et al., 1975). Hence, take as an example a sequence of phonemes *l o f o d u*. According to the EPAM model of phoneme (chunk) learning, a time of 400 ms would be needed to encode each phoneme of that sequence (see Jones, 2012). This means that encoding the sequence *l o f o d u* would require a time of more than 2 s (i.e., 6 × 400 ms), and hence would not be reliably stored in working memory. If, however, the sequence *l o f o d u* is learned by chunking the sequence into adjacent phonemes, *lo fo du*, and if we assume that an additional 30 ms is needed to process each phoneme within a chunk (excluding the first phoneme; see Jones, 2012) less than 2 s (3 × 430 ms) would be needed to process the sequence. Jones (2012) argues that chunking leads to the false perception that short-term memory capacity increases across development: instead, he asserts that it is not capacity that increases across development but the size of chunks (with the use of larger chunks leading toward apparently higher capacity). In an attempt to demonstrate this, Jones used the EPAM model to simulate earlier developmental work on non-word repetition learning. In his simulation, the model was trained on linguistic input (e.g., the non-word *hampent*) while holding capacity and processing speed constant. Over time, the model learned chunks of phoneme sequences. Early in training, many small chunks were extracted from the non-word (e.g., *ha, m, pe, nt*), matching repetition performance of the younger children. Late in training, however, the model extracted a few larger chunks (e.g., h, amp, ent), matching performance of the older children. This illustrates that chunking may offer an important explanation for developmental changes in task performance that involves learning novel word-forms (even when controlling for developmental changes in capacity and processing speed).

According to our working hypothesis, learning within the Hebb repetition paradigm establishes new chunks that are enhanced in memory by subsequent repetitions (Page and Norris, 2009a,b; Page et al., 2013). Jones' (2012) findings let us further hypothesize that encoding of chunks within the sublexical Hebb task takes place at different grain sizes in children compared with adults, with children using a larger number of *small chunks* and adults using a smaller number of *large chunks*. If a Hebb sequence, such as *ja ve ri ka be ti so mu*, is chunked in four two-item chunks as *jave rika beti* and *somu* (note that when chunking this way, the list can be more reliably encoded in working memory, i.e., 4 × 490 = 1960 ms which is below Jones' presumed capacity of 2 s), four chunks will engage for learning on the first presentation of that sequence. As long as the filler sequences are from a different syllable set, there should be nothing to interrupt that learning. If, however, the fillers are made up of the same set (i.e., in the overlap condition), it is likely that anagrams of these chunks, e.g., veja kari tibe muso, will turn up quite often in the non-repeating filler sequences^4^, and hence slow down learning (i.e., an overlap effect as modeled by Page and Norris, 2009b). If on the other hand the same Hebb sequence is chunked into a few larger units, let us say two chunks of four items, *javerika* and *betisomu*, the probability that a full anagram of that chunk (e.g., *vejakari)* turns up early in the filler trials is relatively low (see the same footnote). Hence, larger chunking of syllable sequences representing new word-forms, could explain why adults did not show an overlap effect in the current experiment.

The aim of the third experiment was, therefore, to investigate whether the absence of an overlap effect in adults in Experiment 2 was indeed due to the use of a different chunk size in adults. To test this hypothesis, we encouraged (through a manipulation of time parameters) a sample of adult participants to group Hebb sequences in small, two-item chunks (e.g., jave rika beti somu), just like we suppose children do. We predicted that an overlap effect would emerge when adults are encouraged to memorize small chunks, in contrast to a group of control adults who were not encouraged to chunk small. Furthermore, we predicted that adults who chunk small would show a larger non-overlapping Hebb learning effect compared with that of the control adults, in line with the larger non-overlapping Hebb-learning effect seen for children in Experiment 2. If the small-chunk group of adults indeed shows an item-overlap effect, and a superior non-overlapping Hebb effect, we will be more secure in concluding that chunking strategy (more particularly, preferred chunk size) drives developmental differences in sublexical verbal Hebb sequence learning.

## Experiment 3

### Participants

In total, 59 participants took part in the experiment. All participants were recruited by means of advertising and were randomly allocated to a Hebb-learning condition with chunking (*n* = 29, mean age 29.72 ± 11.33_*SD*_, 20F/9M) or without chunking (*n* = 30, mean age 28.86 ± 12.13_*SD*_, 21F/9M). All participants were living or working in the French part of Belgium. Two participants (one in each condition) were living and working in the Flemish part of Belgium but had a good understanding of the French language. None of them suffered from any developmental, psychiatric or neurological disorders. All participants gave informed consent, and the experiment was approved by the Faculty of Psychology Ethics Commission of the Université catholique de Louvain.

### Materials and procedure

The same materials and procedure were used as in Experiment 2. For the Hebb learning condition *with* chunking, however, no interstimulus interval was provided except after CV_2_, CV_4_, CV_6_, and CV_8_ for which the interval was 1000 ms. These spacing parameters were designed to encourage the participant to chunk the sequence in four two-items chunks, and one one-item chunk (i.e., CV_1_CV_2_ CV_3_CV_4_ CV_5_CV_6_ CV_7_CV_8_ CV_9_). After each sequence, explicit recall was required by use of a recall screen. On the recall screen, presented immediately after presentation of the last CV, the nine CVs were arranged randomly in a circle around a central question mark. Participants were required to recall the CVs in the same order as they were presented by clicking with the mouse device on the syllable. Participants received no cue for clicking, so that a given CV could be clicked more than once. In contrast to the response format used in Experiments 1 and 2 (in which participants had to respond out loud), the recall method did not allow intrusion of CVs that were not presented. The participants were instructed to click the question mark in order to indicate that a CV was omitted in their response. They were told that it would take the position in the sequence where the CV occurred. After each trial, the spacebar was pressed to start the next trial. Note that the positioning of the CVs around the question mark was random on each trial, preventing Hebb learning from being confounded by the learning of a spatial-clicking pattern. All CVs were recorded by a new female voice and presented auditorily at 60 dB using Bose QC 15 headphones. The experiment was presented using a Dell PC running software written in E-prime 2.0.

### Results

**Figure 6:**
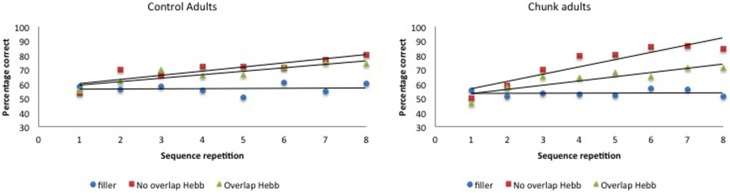
**Performance (percentage of correct scores) as a function of Sequence type (filler vs. Hebb non-overlap vs. Hebb overlap) and Sequence repetition (1–8) in the group with chunking and the control group, Experiment 3. Left panel:** performance for control adults. **Right panel**: performance for chunk-encouraged adults.

McKelvie scoring was used to obtain immediate serial recall scores. The data are plotted in Figure 6.

**Figure 7:**
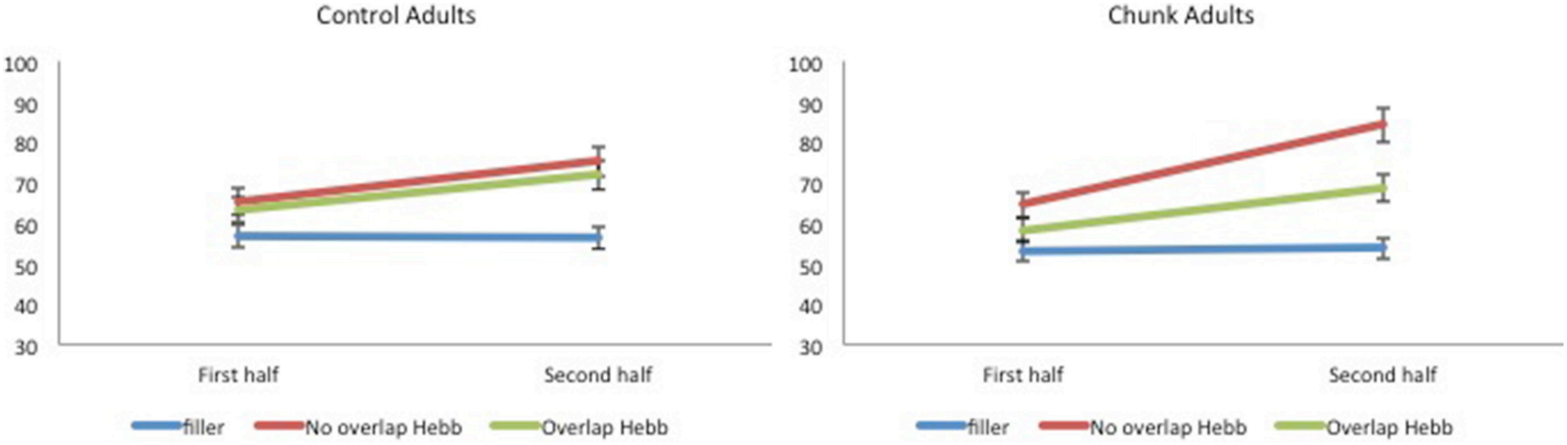
**Mean percentage of items correctly recalled (with standard errors) for Hebb and filler sequences by sequence Halves, in both control and chunk adults, Experiment 3. Left panel:** performance for control adults. **Right panel**: performance for chunk adults.

For each participant, the percentage correct scores were averaged across the first four and last four sequence repetitions, so as to obtain two scores, one for each half, and scores were transformed using arcsin square root transformation. The transformed scores were entered into a 2 (Group: chunk adults vs. control adults) × 2 (Half: first vs. second) × 3 (Sequence type: filler vs. Hebb non-overlap vs. Hebb overlap) repeated measures ANOVA. This yielded no significant effect of Group [*F* < 1]. There was a significant main effect of Half [*F*_(1, 57)_ = 78.74, *p* < 0.001, $n_{p}^{2}=0.58$] such that recall scores for the second half of the repetitions was higher than recall scores for the first half of the repetitions (68.39 ± 1.39_*SE*_ vs. 60.22 ± 1.07_*SE*_), and a significant main effect of Sequence type [*F*_(2, 114)_ = 52.16, *p* < 0.001, $n_{p}^{2}=0.48$]. Comparisons revealed better recall for the non-overlapping Hebb sequence (72.29 ± 1.67_*SE*_) compared with the filler sequences (55.07 ± 1.05_*SE*_)[*F*_(1, 114)_ = 259.42, *p* < 0.001, $n_{p}^{2}=0.69$], and better recall for the overlapping Hebb sequence (65.53 ± 1.48_*SE*_) compared with the filler sequences [*F*_(1, 114)_ = 90.43, *p* < 0.001, $n_{p}^{2}=0.44$]. There was also a significant difference between the two Hebb sequences [*F*_(1, 114)_ = 43.52, *p* < 0.001, $n_{p}^{2}=0.28$]. Crucially, there was a significant interaction between Half and Sequence type [*F*_(2, 114)_ = 29.18, *p* < 0.001, $n_{p}^{2}=0.45$], that in turn interacted significantly with Group [*F*_(2, 114)_ = 4.09, *p* < 0.05, $n_{p}^{2}=0.16$]. This three-way interaction is illustrated in Figure 7. Planned comparisons within both groups revealed a significant non-overlapping Hebb effect (i.e., a different improvement across halves for filler and non-overlapping sequences) for chunk adults [*F*_(1, 114)_ = 50.56, *p* < 0.001, $n_{p}^{2}=0.31$], and control adults [*F*_(1, 114)_ = 12.92, *p* < 0.01, $n_{p}^{2}=0.10$]. This non-overlapping Hebb effect was significantly larger for chunk adults compared with control adults [*F*_(1, 114)_ = 6.51, *p* < 0.05, $n_{p}^{2}=0.05$]. Further comparisons revealed the presence of an overlapping Hebb effect in both chunk adults [*F*_(1, 114)_ = 10.86, *p* < 0.01, $n_{p}^{2}=0.09$] and control adults [*F*_(1, 114)_ = 9.80, *p* < 0.01, $n_{p}^{2}=0.08$]. This did however not differ between groups, *F* < 1. Chunk adults showed a significant lower improvement across halves for the overlapping Hebb sequence compared with the non-overlapping Hebb sequence [*F*_(1, 114)_ = 14.56, *p* < 0.001, $n_{p}^{2}=0.11$]. There was no such difference for control adults, *F* < 1. Chunk adults and control adults did not differ on differences across halves for the filler sequences, *F* < 1. During the first half of the task, both groups showed significantly better recall for the non-overlapping Hebb sequence compared with the filler sequence [control, *F*_(1, 114)_ = 15.63, *p* < 0.001, $n_{p}^{2}=0.12$; chunk, *F*_(1, 114)_ = 21.26, *p* < 0.001, $n_{p}^{2}=0.16$], and for the overlapping Hebb sequence compared with the filler sequence [control, *F*_(1, 114)_ = 8.66, *p* < 0.01, $n_{p}^{2}=0.07$; chunk, *F*_(1, 114)_ = 4.07, *p* < 0.05, $n_{p}^{2}=0.03$]. The chunk group also showed significantly better recall for the non-overlapping Hebb sequences compared with the overlapping Hebb sequence, *F*_(1, 114)_ = 6.73, *p* < 0.05, $n_{p}^{2}=0.06$]. For all contrasts, there were however no differences between groups, *Fs* < 1. During the second Half of the task, only the chunk group showed better recall for the non-overlapping Hebb sequence compared with the filler sequence, *F*_(1, 114)_ = 63.86, *p* < 0.001, $n_{p}^{2}=0.36$]. During the second half of the task, the non-overlapping Hebb effect (i.e., difference between Hebb and filler sequence) was significantly higher for the chunk group compared with the control group [*F*_(1, 114)_ = 17.01, *p* < 0.001, $n_{p}^{2}=0.13$].

### Discussion

In Experiment 3, the same sublexical material was used as in Experiment 2. One sample of adult participants (i.e., the chunk adults) was, however, encouraged to group Hebb and filler sequences in small, two-item chunks (e.g., *jave rika beti somu*). While the control adults again showed no effect of item-overlap, replicating the null effect for adults in Experiment 2, adults that were encouraged to chunk small did show a reliable item-overlap effect, similar to the children in Experiment 2. Indeed, small-chunk adults showed less improvement on the overlapping compared with the non-overlapping Hebb sequences. Moreover, we observed a larger (non-overlapping) HRE in adults that were encouraged to chunk small compared with the control adults that were not encouraged to do so. Note that recall during the first half of the task was higher for Hebb sequences compared with filler sequences. This again indicates a rapid memorization of the Hebb sequence during the first four repetitions (see Figure 6). Chunking in particular helped rapid learning of the non-overlapping Hebb sequence (as reflected by better recall for the non-overlapping Hebb sequence during the first half of the task, only in the chunk group).

## General discussion

Words are essentially sequences of smaller, sublexical constituents (i.e., phonetic features, phonemes, syllables) that combine to make larger lexical representations (Pinker, 1994). In order to learn such a (complex) combinatorial set, children must be able to isolate starting elements from the sequential input they are exposed to and then gradually acquire the pattern of legal combinations (Newport et al., 2004). Although children are commonly believed to be better language learners than adults, it remains to this day unclear whether or how children and adults differ in terms of the serial-order learning mechanisms that underlie novel word-form learning. In the present work, we investigated serial-order learning differences between children and adults, using a laboratory analog of novel word-form acquisition, better known as the HRL effect. In a first experiment, sequences of existing words were presented for immediate serial recall. One of the repeating Hebb sequences contained the same words as the filler sequences (the overlap condition). We found comparable HREs in both children and adults. Moreover, we found reduced Hebb learning due to overlap in adults, replicating previous studies, and, for the first time, the same effect was also observed in children. One limitation regarding this experiment and previous studies, though, concerns the use of lexical items (words) in the sequential input. Sequences of lexical items are not equivalent to novel words, which are essentially sequences of sublexical items, and the word sequences might therefore have obscured potentially stronger learning effects in children. To address this question, a second experiment was designed to compare children and adults on a Hebb repetition-learning task using sublexical sequences mimicking novel words. Importantly, we found that children now showed a stronger HRE compared with adults. Surprisingly, however, only children showed reduced learning due to item-overlap between Hebb and filler sequences. This is a very interesting finding, particularly if we assume that children learn new word-forms by chunking them in smaller units than do adults (Jones, 2012). Small two-syllable chunks in Hebb sequences (e.g., AB CD EF GH) are more sensitive to item-overlap than larger, three or four-syllable chunks (e.g., ABCD EFGH). With reference to the chunk-learning account of Page and Norris (2009b), it is more likely that a perfect anagram of a small chunk shows up in the filler sequences, slowing down HRL, compared to a perfect anagram of a larger chunk, which is assumed to have a less detrimental effect on Hebb learning. This was tested more directly in a third experiment in which we encouraged adults to chunk sublexical sequences smaller, i.e., into four two-syllable units. This resulted in the appearance of an item-overlap effect and most importantly, it improved HRL in a similar manner as we observed in children.

The notion of starting small has already been proposed within word-learning theories (Elman, 1991, 1993) and was also supported by subsequent empirical studies (Conway et al., 2003). This gave rise to the less-is-more hypothesis in language learning (Newport, 1990). Newport explained that children are better able to learn languages than adults *because* they have fewer cognitive resources available (smaller working memory capacities). Children will naturally proceed by beginning with small parts and will proceed to more complex constructions as they mature. More competent adults will begin by trying to acquire larger structures from the start because their cognitive resources allow them to do so. Interestingly, Jones (2012) proposes an alternative view in which he argues that chunking (or starting small) should be considered as an explanation for developmental differences in cognitive behavior *without* the need for additional developmental changes in short-term memory capacity or processing speed. In his view, changes in short-term memory capacity can more likely be seen as the consequence, rather than the cause of changes in chunk behavior. According to Rohde and Plaut (2003), starting small is, by itself, not a critical condition to reach linguistic fluency and is only beneficial for children because their learning is characterized by a (connectionist) system that is still unorganized and inexperienced yet still highly flexible to future adaptation, in contrast to that of adults. This also accords with the granularity effect that has been described within the grammar-learning domain (Arnon and Ramscar, 2012). Arnon and Ramscar showed that, during grammatical gender learning (i.e., learning new article + noun combinations), adults benefited more from exposure to the full complex sentence before exposure to the single nouns. Similarly, in the current study, we found that small chunking was beneficial for HRL but only when there was no full item-overlap between sequences. Item-overlap causes strong competition from interfering structures in the filler sequences making Hebb learning difficult. This suggests that for complex linguistic input, other more adult-adapted learning strategies are necessary. We assume that chunking sequences in larger units is one of those strategies. It explains the lack of an overlap-effect in the non-word Hebb task because it is less likely that anagrams of a large chunk show up in the filler sequences.

What is still not clear from the current study is whether children and adults, if anything, use a different grain size for chunking the lexical Hebb sequences. In Experiment 1, when lexical sequences were presented for immediate recall, children and adults both showed a comparable item-overlap effect. There are two chunking sizes that could explain this item-overlap effect. Either, both children and adults chunk lexical Hebb sequences in small two-word units for which competing anagrams turn up quite often in the filler sequences, or, adults represent the entire lexical Hebb sequence as one large chunk that receives competition from its perfect anagram in every filler sequence (the same sequence but in a different order—this was the explanation originally offered by Page and Norris, 2009b). According to Jones (2012) chunking depends on the amount of exposure to the stimuli in the environment (prior knowledge): more exposure leads to larger chunks. Recently, it has been found that prior learning of item-by-item transitions affects immediate recall of word sequences (e.g., *chou feu veau pain*, etc.) and non-word sequences (e.g., *chon zin bi leuh*, etc.) (Majerus et al., 2012). In contrast, immediate recall of digit sequences is only affected by prior learning of the entire sequence. The authors argue that digits are linguistic chunks that we frequently experience in large arbitrary combinations (e.g., phone numbers) while this is not the case for sequences of random words or non-words. This results in the false perception that short-term memory “capacity” for digits is superior to short-term memory for words. With this in mind, we might assume that children vs. adults use different chunking strategies for lexical vs. sublexical sequences, resulting in different competition effects in Experiments 1 and 2. This could be a reflection of underlying differences in experience with the sequential input, independently of potential differences in working memory capacity. Future research should shed more light on the dissociation between working memory capacity and prior knowledge as possible factors driving developmental sensitivities in Hebb learning.

## Conclusion

Why are children better language learners than adults? This is an important question in the light of the sensitive-period theory of language acquisition. The current study approaches this question from a memory and learning perspective in which we assume that children are better language learners because they chunk linguistic structures in smaller subsequences compared with adults (a species of the *less-is-more* hypothesis). Previous studies showed that HRL, a sequential learning analog of word-form learning, is rather weak in children. This is not in accordance with the sensitive-period hypothesis, according to which we would predict strong Hebb learning effects in children. The lack of strong Hebb learning effects in children in previous studies is likely to be explained by (a) the use of stimulus materials that do not resemble naturalistic word learning (i.e., sequences of words or digits instead of sequences of syllables or phonemes), and (b) the item-overlap between sequences that results in weaker Hebb learning, at least in adults. The current study was the first to test these hypotheses by directly comparing children and adults on a Hebb-learning task that contains either lexical or sub-lexical sequences, and with or without item-overlap. Furthermore, children and adults' Hebb-learning differences were directly assessed within the less-is-more hypothesis of language acquisition by encouraging adults to chunk Hebb sequences into small units. Overall, we found that children (Experiment 2) and small-chunking adults (Experiment 3) showed superior HRL performance. This suggests that children and adults differ in the way they chunk verbal sequential material, potentially offering insights into the sensitive-period hypothesis for language acquisition. Most importantly, the present study shows that human-memory theories have a significant potential to improve our understanding of the cognitive processes that lay the foundation of language acquisition across life.

## Author contributions

ES contributed to conception and operationalization of the study as well as the acquisition, analysis and interpretation of the data, and the writing of the manuscript. LB contributed to the conception of the study, the interpretation of the data and the content/editing of the manuscript. MS contributed to the operationalization of the first two experiments as well as their data acquisition. MP contributed to the conception of the study, the interpretation of the data and the content/editing of the manuscript. WD contributed to the conception of the study and the content/editing of the manuscript. ME contributed to the content/editing of the manuscript. AS contributed to the conception and operationalization of the study as well as the interpretation of the data and the content/editing of the manuscript.

## Funding

This work was facilitated by a grant from the Fonds de la Recherche Scientifique - FRS – FNRS (Belgium), grant “Crédit aux Chercheurs 2013-2015 1.A.915.14F,” to the first author.

### Conflict of interest statement

The authors declare that the research was conducted in the absence of any commercial or financial relationships that could be construed as a potential conflict of interest.

## Footnotes

^1^Both native and non-native French speakers were included. All participants used French on a daily base (i.e., in school or at work).

^2^For example, a sequence such as “bras pied rue boue oie pluie jour nuit” could be recalled as “bras pied rue oie pluie nuit.” This response would be scored as follows: 3 correct in the first step (i.e., “bras pied rue” from left to right); 1 correct in the second step (i.e., “nuit” from right to left); 2 correct in the third step (i.e., “oie pluie” occur together); and 0 in the last step (i.e., no other items in the correct position), for a total score of 6 (3 + 1 + 2 + 0) out of 9.

^3^We present the first/second half analysis for matters of comparability with earlier studies on Hebb learning in developmental samples (e.g. Mosse and Jarrold, 2008; Archibald and Joanisse, 2013). The same analysis with regression slopes as a measure of Hebb repetition learning yielded qualitatively similar results. The entire dataset (i.e. with regression measures and with first/second half measures) can be downloaded at: https://github.com/NOORES/Chunking-in-Children-and-Adults/commit/c119681411d646c57a17efe10067c3139674f701.

^4^The probability that a syllable *ve* turns up in a random filler sequence at an odd position is 0.5, and in 1/7 of these occasions there will be a *ja* following this syllable, giving it a 1/14 chance that *veja* turns up as a learnable chunk in the filler sequence. The same applies for each of the other chunks, giving a probability of around 0.29 that a random filler sequence contains a chunk that is a perfect anagram of one of the chunks in the Hebb sequence. In contrast, when chunking Hebb sequences in larger, say two 4-item, chunks, the probability that any combination of *ja ve ri* and *ka* (other than *javerika* itself) occurs as a chunk in either half of a filler sequence would be only 0.027. So if you chunk the Hebb sequence in fours it is relatively unlikely that you will get perfect anagrams of those chunks in similarly chunked filler sequences, thus minimizing the extent to which learning is slowed by item overlap between Hebb sequences and fillers.
